# Janus Poly(Vinylidene Fluoride) Membranes with Penetrative Pores for Photothermal Desalination

**DOI:** 10.34133/2020/3241758

**Published:** 2020-03-03

**Authors:** Hao-Hao Yu, Lin-Jiong Yan, Ye-Cheng Shen, Si-Yu Chen, Hao-Nan Li, Jing Yang, Zhi-Kang Xu

**Affiliations:** MOE Key Laboratory of Macromolecular Synthesis and Functionalization, and Key Laboratory of Adsorption and Separation Materials & Technologies of Zhejiang Province, Department of Polymer Science and Engineering, Zhejiang University, Hangzhou 310027, China

## Abstract

Solar-driven desalination has been considered as a promising technology for producing clean water through an abundant and pollution-free energy source. It is a critical challenge to reasonably design the porous morphology and the thermal management of photothermal membranes for enabling efficient energy conversion and water production. In this work, a Janus poly(vinylidene fluoride) membrane was fabricated in combination of penetrative pore structure, asymmetric surface wettability with proper thermal management for high-efficiency solar desalination. Highly open and directly penetrative pores achieved by the two-dimensional solvent freezing strategy are considered to provide direct pathways for water and vapor transportation. The unique feature of hydrophobic upper layer/hydrophilic lower layer enables the photothermal membranes to self-float on the water surface and rapidly pump water from the bulk to the surface. The resulting Janus membrane exhibits a satisfactory light absorbance as high as 97% and a photothermal conversion efficiency of 62.8% under one-sun irradiation in a direct contact mode. The solar-to-vapor efficiency rises up to 90.2% with the assistance of a thermal insulator adopted beneath. Both the Janus membrane and the composite setup are able to work efficiently with a high stability in seawater desalination, and the concentration of ion in condensed water is reduced to below 1 ppm. Therefore, Janus membranes with directly penetrative pores and photothermal surfaces shine a light on the development of high-performance solar evaporators for the practical application in solar seawater desalination.

## 1. Introduction

Freshwater scarcity is one of the most urgent issues for human beings in modern society. Solar-driven evaporation for clean water production is currently considered as an effective solution to alleviate water shortage because of the ecofriendly and inexhaustible solar energy [1, 2]. Self-floating evaporators with a highly efficient energy conversion and fresh water production have attracted intensive attention in the solar desalination technology due to their strong localization effect on heat conduction at air/water interface [3–10].

Generally, the design philosophy of self-floating solar-driven evaporators with a high efficiency emphasizes an integrative optimization of light absorption, thermal management, and water transportation. First, a high-performance solar steam generation strongly relies on the photothermal materials to absorb sunlight as much as possible. Carbon-based materials (*e.g.*, reduced graphene oxide [11, 12], carbon nanotubes [13], carbon black [14, 15]), plasmonic nanoparticles [16, 17] (*e.g.*, Al or Au), and super-black polymers (*e.g.*, polypyrrole (PPy) [18, 19], polydopamine (PDA) [20]) have been extensively adopted due to their remarkable solar-thermal conversion efficiency. Second, an elaborated thermal management is required, aiming to confine the absorbed heat on the water surface and then to ensure thermal insulation from the bulk water. It can be achieved using the evaporator materials with low density [21] and hydrophobicity [22] or with the aid of porous foams [23, 24]. The floating status of the evaporators on the water surface suppresses the unexpected heat conduction downward to the bulk water. Third, a rapid water and vapor transportation is preferential to facilitate the water production efficiency, including water pumping from the bulk body to the evaporation surface *via* the capillary effect and steam escape from the photothermal surface to the condenser [25]. To this end, delivery channels with a low mass transfer resistance should be a significant consideration for solar evaporators. Porous substrates containing highly open channels [26] with suitable hydrophilicity [27] play a vital role in these concerns above, because they work as not only supports for photothermal materials but also channels for water and vapor transfer. Emerging membranes with directly penetrative pores across the membrane thickness are preferential substrates for the fabrication of self-floating solar evaporators. For instance, a solar evaporator made from natural wood achieves a light absorbance of around 99% due to the waveguide effect of columnar pores incorporated with plasmonic nanoparticles [28]. Meanwhile, its micro- and nanochannels with a low tortuosity can efficiently pump water from the bottom of the device because of the capillary wicking effect [28]. Similarly, anodic aluminum oxide (AAO) membranes loaded with Al plasmonic nanoparticles on their nanochannel walls feature directly penetrative pores for water delivery. These pores also function as efficient light trappers to achieve an impressive light absorbance of 99% [17]. More recently, auricle-inspired 3D photothermal cones are developed for high-efficiency solar-driven evaporation. Commercial hydrophilic polyvinylidene fluoride (PVDF) membranes are deposited with PPy and then folded into cones to minimize light reflection and heat loss to bulk water. They exhibit a high solar absorbance of 99.2% which is higher than that of the plane membranes (93%) due to the multiple reflection of sunlight within the 3D cones [18]. Nevertheless, the limited processability is an intractable issue for natural wood. AAO membranes usually suffer from low porosity and mechanical fragility, and their preparation process may impose a restriction on the large-scale fabrication. Moreover, the fabrication of 3D cones involves complicated and multistep processes, including folding and bonding the membrane and sealing the hole on the apex. Besides, the surface area of 3D cores is much larger than the projected area, which could lead to a high consumption of photothermal materials. Hence, it is highly required to develop easy-to-modify and easy-to-process membranes with directly penetrative pores, high porosity, and mechanical robustness for solar-driven photothermal evaporators with high performances.

**Figure 1:**
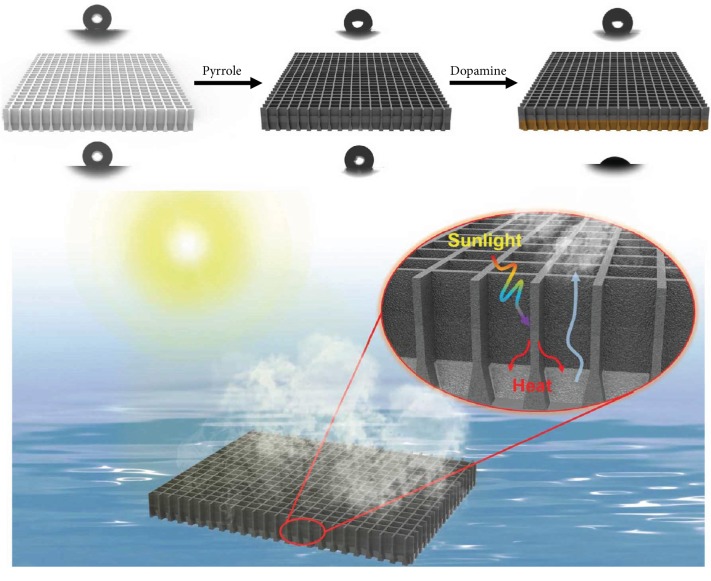
Schematic illustration to the fabrication of Janus VOPM for photothermal desalination.

In our previous work, vertically oriented porous membranes (VOPMs) were prepared *via* a thermally induced phase separation process [29]. The growth of solvent crystal was controlled from one side to the other side within the PVDF membrane through a well-designed bidirectional freezing process. Micron-sized penetrative pores were thus formed across the membrane thickness after removing solvent crystals by extraction or sublimation. The resulting VOPMs possess a high porosity (70-80%), and their directly penetrative pores feature large openings at one side and small ones at the other side, like cones neatly inserted across the membrane. Such distinctive porous morphology makes VOPMs an ideal base substrate for interfacial solar evaporators. First, directly penetrative pores are conducive to water pumping and steam escape. Second, the cone-like pores are able to fully absorb solar energy due to the multiscattering effect. Herein, hydrophobic PPy was coated on the inner pore walls of VOPM *via* chemical vapor deposition polymerization (CVDP) [30]. Hydrophilic PDA was further deposited on the single side of the PPy-coated VOPM to construct a Janus structure (Figure 1) *via* the rapid oxypolymerization of dopamine according to our previous work [31, 32]. The resulting Janus VOPM floats on the water surface with the hydrophobic layer towards air for absorbing sunlight irradiation and the hydrophilic channels facing water for pumping water and delivering steam (Figure 1). Such Janus structure results in a light absorption capacity as high as 97% and an accelerated water evaporation rate up to 1.08 kg·m^−2^·h^−1^ under one-sun irradiation in a direct contact mode. The final solar-to-vapor conversion efficiency is 62.8%. Furthermore, with the aid of a thermal insulator and a 2D water path [23] settled beneath the Janus VOPM, the evaporation rate rises to 1.58 kg·m^−2^·h^−1^ and the conversion efficiency reaches up to 90.2%. This composite device is able to operate smoothly in seawater desalination for several hours, and the content of inorganic ions in condensed water is reduced to below 1 ppm. This study provides a new insight in designing highly efficient solar evaporators and holds promise for scale-up desalination because of the simple membrane fabrication with high scalability.

## 2. Results and Discussion

**Figure 2:**
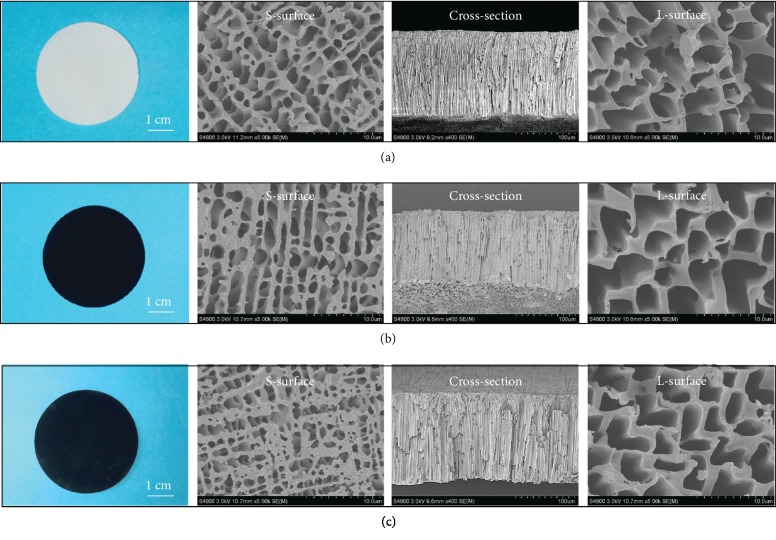
Digital photos and scanning electron microscopy (SEM) images of (a) nascent VOPM, (b) PPy-coated VOPM, and (c) Janus VOPM.

The fabrication process of VOPMs is well controlled on the basis of our previously proposed two-dimensional solvent freezing strategy [29]. Dimethyl sulfone (DMSO2) is a good solvent for PVDF at a high temperature. It is able to crystallize within the polymer matrix in a designed cooling process with two-dimensional temperature gradients. The nucleation of DMSO2 is caused by the addition of cooling water. Meanwhile, the temperature gradient originated from the difference in thermal conductivity between stainless steel and glass promotes the crystal growth along the direction from the stainless-steel plate to the glass one. The closer the precursor solution contacts the glass plate, the more crystallization time is available to form large crystals in the cooling process, producing cone-like DMSO2 crystals with the tapered tip towards the stainless-steel plate. After removing DMSO2 crystals by extraction, cone-like and directly penetrative pores are generated in the thickness direction of the PVDF membrane. The membrane surface contacting the stainless-steel plate shows small pores (diameter ≈ 1 *μ*m) while the other surface touching the glass plate displays large pores (diameter ≈ 3 *μ*m) (Figure 2(a)). The corresponding surfaces are denoted as S-surface and L-surface, respectively. The as-prepared VOPMs were further used as the skeletons to fabricate photothermal membranes with a homogeneous PPy coating on inner pore walls *via* a facile CVDP process [30]. The white nascent VOPM (Figure 2(a)) becomes black (Figure 2(b)) after PPy coating. To further impart the PPy-coated VOPM with hydrophilicity, PDA was decorated on the S-surface using the single-side-floated deposition triggered by CuSO_4_/H_2_O_2_ [31]. The resulting Janus VOPM exhibits a brown side after PDA coating (Figure 2(c)). It is noteworthy that both postmodification processes have no influence on the pore morphology of VOPM, still maintaining an open porous structure without blockage (Figures 2(b) and 2(c)). The chemical structure of each decorated layer was analyzed by attenuated total reflection Fourier transform infrared spectroscopy (ATR-FTIR) ([Supplementary-material supplementary-material-1]) and X-ray photoelectron spectroscopy (XPS) ([Supplementary-material supplementary-material-1], [Supplementary-material supplementary-material-1]). The decoration of VOPM is also reflected by the variation of surface wettability. The water contact angles (WCA) of nascent VOPM and PPy-coated one are >120° due to the inherent hydrophobicity of PVDF and PPy ([Supplementary-material supplementary-material-1]). After the deposition of PDA, the WCA of S-surface decreases to <90° while that of L-surface remains >120°, verifying an asymmetric wettability for the Janus VOPM. Moreover, the WCA of S-surface shows a continuous decline with increasing the deposition time ([Supplementary-material supplementary-material-1]), suggesting an increasing coverage of PDA on the surface of pore walls to achieve a hydrophilization on the single side of VOPM. The hydrophilization depth detected by laser scanning confocal microscopy (LSCM) [33] is about 15 *μ*m at a deposition time of 20 min and shows no obvious increasing tendency with prolonging the time ([Supplementary-material supplementary-material-1]).

**Figure 3:**
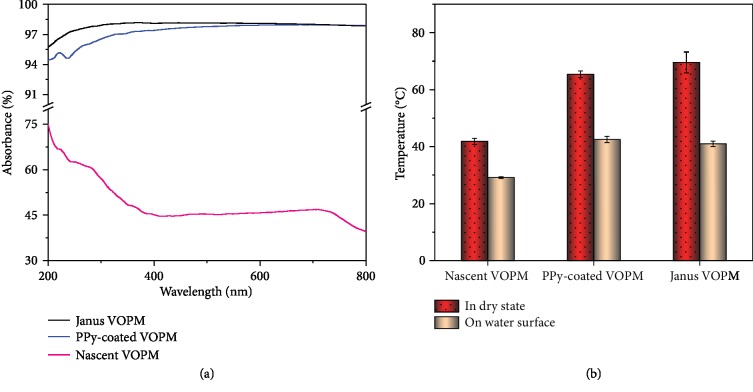
Light absorption capacities of various VOPMs. (a) UV-Vis absorption spectra and (b) equilibrium surface temperatures of nascent VOPM, PPy-coated VOPM, and Janus VOPM in a dry state and in a floating state under one-sun irradiation.

The solar absorption ability of photothermal materials plays a predominate role in the efficiency of the solar vapor generation process. The solar absorbance (*A*) of membrane under one-sun irradiation is calculated by Equation (1):

(1) $$\begin{array}{c} A=\left(1-R-T\right)\times 100\% \end{array}$$

where *T* is the transmittance and *R* is the diffuse reflectance measured from the UV-Vis spectra in the wavelength range of 200-800 nm ([Supplementary-material supplementary-material-1]). The solar absorbance of nascent VOPM is maintained at a relatively low level (40%-45% in the visible light range and 45%-70% in the UV region) while that of PPy-coated VOPM reaches >94% in the full range of wavelength (Figure 3(a)). It further increases up to ~97% after the PDA deposition on the S-surface (Figure 3(a)). Such significantly enhanced solar absorption is ascribed to the deposition of light absorbers (PPy and PDA) onto the pore walls of VOPM. Besides, it is notable to see that L-surface exhibits a stronger absorption ability than S-surface under solar irradiation for the VOPM homogeneously coated with PPy ([Supplementary-material supplementary-material-1]). This phenomenon can be explained by the stronger antireflectivity of large pores compared with small ones under a simulated sunlight with the same intensity ([Supplementary-material supplementary-material-1]). L-surface was, therefore, chosen to be the surface towards sunlight for the solar-driven evaporation in this work. To assess the light-to-heat ability, the surface temperatures of photothermal membranes were probed by a thermal infrared camera. The surface temperatures of Janus VOPM and PPy-coated VOPM in a dry state are 69.6 ± 3.7°C and 65.4 ± 1.2°C under one-sun irradiation, respectively, which are much higher than that of nascent VOPM (41.9 ± 1.1°C) (Figure 3(b)). In a floating state, both Janus VOPM (41.0 ± 0.9°C) and PPy-coated VOPM (42.5 ± 1.1°C) also exhibit higher surface temperatures than the nascent VOPM (29.6 ± 0.3°C) (Figure 3(b)). These results predict the superiority of Janus VOPMs in interfacial heating for the solar steam generation.

**Figure 4:**
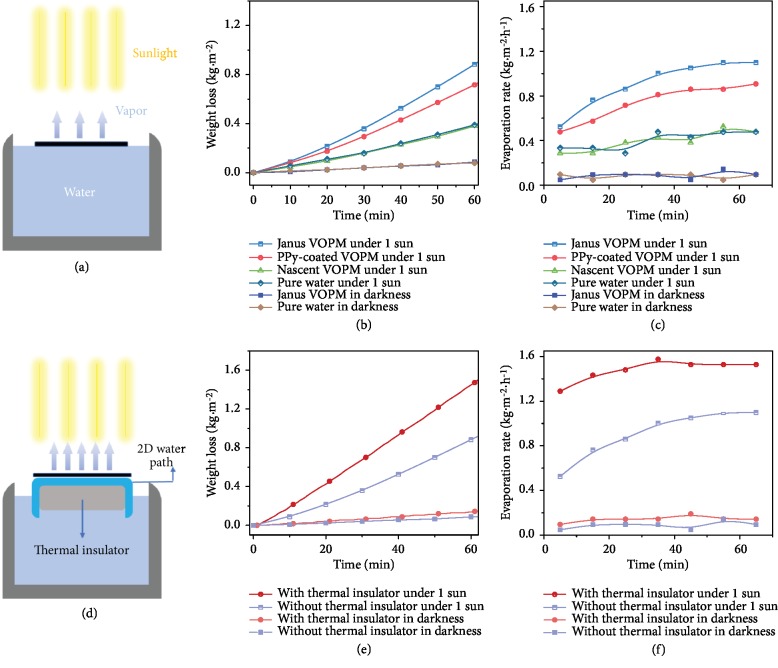
Setups and their performances of solar vapor generation. (a) Direct contact mode for solar evaporation. (b) Weight loss and (c) evaporation rate of water as a function of irradiation time using different VOPMs in a direct contact mode. (d) Composite device equipped with a thermal insulator and an absorbent paper for solar evaporation. (e) Weight loss and (f) evaporation rate of water as a function of irradiation time using Janus VOPMs with or without the thermal insulator.

To investigate the performance of solar vapor generation, different VOPM samples were placed onto the water surface under a simulated solar source which is vertically placed above the membrane (Figure 4(a)). The weight loss of water during the solar evaporation was recorded in real time with a balance below the beaker ([Supplementary-material supplementary-material-1]). Pure water evaporation under one-sun irradiation and in darkness were adopted as controls. All VOPM samples are able to self-float on the water surface benefiting from the hydrophobicity of PVDF and PPy coatings. The evolutions of water weight loss and corresponding evaporation rate over irradiation time were recorded in Figures 4(b) and 4(c), respectively. Under one-sun irradiation, the water evaporation rate of nascent VOPM slightly increases over time and reaches 0.48 kg·m^−2^·h^−1^ after 65 min. This behavior is consistent with the natural water evaporation under the same condition, suggesting incapability of nascent VOPM to convert light to heat. In contrast, both Janus VOPM and PPy-coated VOPM significantly accelerate water evaporation with an ever-increasing rate over time. Their evaporation rates are 1.10 kg·m^−2^·h^−1^ and 0.91 kg·m^−2^·h^−1^ after 65 min, respectively. The relatively rapid water evaporation using Janus VOPM is attributed to the hydrophilized water transportation pathway coated with PDA which speeds up the water delivery during the solar evaporation. It is worthy to note that the hydrophilization depth of Janus VOPM remains constant with prolonging the deposition time ([Supplementary-material supplementary-material-1]) as mentioned above. This result provides a reasonable explanation for the stable water evaporation rates achieved by Janus VOPMs with different deposition time ([Supplementary-material supplementary-material-1]). As a control, the evaporation rate of Janus VOPM in darkness is almost invariable over time until it reaches 0.10 kg·m^−2^·h^−1^ after 65 min, which is as same as the pure water evaporation (Figures 4(b) and 4(c)). The solar-to-vapor conversion efficiency (*η*) was further calculated by Equation (2) [3]:

(2) $$\begin{array}{c} \eta =\frac{\left(\dot{m}-{\dot{m}}_{0}\right)H_{v}}{C_{\text{opt}}P_{0}} \end{array}$$

where $\dot{m}$ is the evaporation rate, $\dot{m_{0}}$ is the natural evaporation rate without irradiation, *H*_*v*_ is the enthalpy of liquid-vapor phase change (~2260 kJ·kg^−1^), *C*_opt_ is the optical concentration, and *P*_0_ is the power density of one-sun irradiation (1 kW·m^−2^).

The solar-to-vapor conversion efficiency of natural water evaporation under one sun is only 23.8%, while those of PPy-coated VOPM and Janus VOPM reach up to 54.6% and 62.8%, respectively. Although this efficiency assisted with Janus VOPM is 2.6 times higher than that of natural water evaporation, it is still much lower than the light absorbance of the membrane (>97%). The reason for this loss of absorbed solar energy is ascribed to the intrinsic limitation of the direct contact mode (Figure 4(a)) in which a significant portion of absorbed energy unavoidably dissipates through the bulk water. To suppress the heat loss, a composite device equipped with a thermal insulator layer and a 2D water path was adopted [23], as illustrated in Figure 4(d). A hydrophobic polymer foam (closed-cell polyurethane foam, thickness ≈ 20 mm, thermal conductivity ≈ 0.034 W·m^−1^·K^−1^) was wrapped by an absorbent paper, and the Janus VOPM was placed on the top. The downward heat conduction can be dramatically reduced by the thermally insulated foam. Meanwhile, water is continuously pumped to the light absorber layer through the absorbent paper. In this way, both efficient water supply and depressed heat loss can be achieved simultaneously. With this device, the equilibrium surface temperature under one-sun irradiation reaches 47.0 ± 0.44°C, much higher than that using the direct contact mode (41.0 ± 0.9°C). Therefore, the evaporation rate strikingly increases up to 1.58 kg·m^−2^·h^−1^ after 35 min and remains relatively stable over time (Figures 4(e) and 4(f)), resulting in an enhancement of solar-to-vapor conversion efficiency up to 90.2 ± 3.1% under one-sun irradiation.

Table 1 summarizes the performances of solar evaporators using recently reported photothermal membranes. The Janus VOPM with penetrative pores in this work achieves a high evaporation rate and solar-to-vapor conversion efficiency compared with others, especially when it is equipped with a thermal insulator. It is also worth stressing that the Janus VOPM shows a higher solar-to-vapor conversion efficiency than membranes with a bicontinuous structure *via* phase inversion [18] and fibrous membranes *via* electrostatic spinning [15]. It is the microsized cone-like pores which are directly penetrative through the membrane thickness that promote the light harvesting and improve the vapor escape rate due to the capillary wicking effect, generating water steam more efficiently.

**Figure 5:**
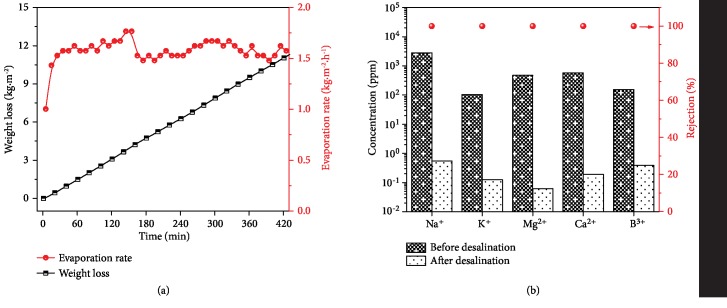
Seawater desalination performances of a Janus VOPM with a thermal insulator. (a) Weight loss and evaporation rate of water as a function of irradiation time under one-sun illumination. (b) Ion concentrations of seawater before and after desalination. Red balls refer to the calculated rejection ratios of ions.

In view of the requirements for effective and efficient solar desalination *via* solar-driven steam generation, the seawater evaporation performances using Janus VOPM were further investigated. With the aid of a thermal insulator, the seawater evaporation remains stable with a rate of 1.5~1.6 kg·m^−2^·h^−1^ throughout a continuous operation for 7 h, indicating a high solar-to-vapor conversion efficiency and a long-term durability for seawater desalination (Figure 5(a)). The stability of the system was further investigated by a cyclic testing in which the operation time for each cycle is 1 h. The evaporation rates are almost invariable during 10 cycles, maintaining a high level of >1.5 kg·m^−2^·h^−1^ ([Supplementary-material supplementary-material-1]). Furthermore, a concentrated NaCl solution (20%) was used to test the performance of solar desalination ([Supplementary-material supplementary-material-1]). The evaporation rate increases at first since the surface temperature is getting higher due to the light-to-heat conversion. However, it shows a decline in half an hour because of the salt crystal deposition on the membrane surface. The asymmetric wettability of the Janus VOPM can effectively prevent the salting-out effect since the salts accumulated onto the hydrophilic PDA layer can be quickly dissolved through the continuous water pumping at the initial stage. However, the salt concentration increases greatly in the 2D water path (absorbent paper), and eventually, the salt crystals are formed at the interface between the Janus VOPM and the absorbent paper in a few hours ([Supplementary-material supplementary-material-1] insert). These salt crystals would intensify the light reflection and block the water or vapor channels, resulting in a depression of evaporation rate ([Supplementary-material supplementary-material-1]). What is more, the accumulated salts would bring a mechanical stress to the device and even damage the Janus VOPM. On the contrary, the Janus VOPM in a direct contact mode without a thermal insulator is able to operate smoothly for as long as 12 h without an obvious decrease in evaporation rate ([Supplementary-material supplementary-material-1]), indicating that the membrane with directly penetrative pores and the Janus structure efficiently prevent the salt blocking. Consequently, we suggest that the Janus VOPM with a direct contact mode on water surface is more suitable to achieve a long-term stability for the solar desalination of highly brackish water. However, for desalination of seawater or of other systems with relatively low salt concentration, the Janus membrane with a thermal insulator is preferred to greatly improve the efficiency of water evaporation.

Eventually, the ion concentrations in seawater and condensed water were measured by inductively coupled plasma emission spectroscopy (ICP) to evaluate the quality of desalinated fresh water through the solar-driven evaporation. The concentrations of all inorganic ions decrease sharply below 1 ppm after the evaporation-condensation process (Figure 5(b)). The rejections to these ions are as high as 99.8%, which is fully compliant with the potable water standard issued by the World Health Organization.

## 3. Conclusion

In summary, we have developed a facile strategy to fabricate Janus photothermal membranes with directly penetrative cone-like pores for solar steam generation. Hydrophobic super-black PPy and hydrophilic PDA are successively coated onto the opposing surfaces of PVDF VOPM to construct both photothermal function and asymmetric wettability. The obtained Janus photothermal membranes show a high light absorption of over 97% and are able to convert sunlight to heat effectively. The water evaporation rate of the Janus VOPM can be accelerated up to 1.10 kg·m^−2^·h^−1^ under one-sun irradiation using a direct contact mode, and the solar-vapor conversion efficiency is calculated to be 62.8%. With the assistance of a thermal insulator, the water evaporation rate of Janus VOPM increases to 1.58 kg·m^−2^·h^−1^ and the solar-vapor conversion efficiency rises to 90.2%. The setup with a thermal insulator is able to operate continuously in seawater, and the contents of salt ions in the condensed water decrease to below 1 ppm. The present strategy provides a way for rational design and controllable construction of high-performance photothermal membranes, showing great potential applications in seawater desalination, waste water treatment, and solution concentration.

## 4. Materials and Methods

### 4.1. Experimental Design

Thermally induced phase separation is used to prepare PVDF membranes with directly penetrative pores *via* a two-dimensional solvent freezing technique. Subsequently, PPy is coated on one surface of the membrane *via* chemical vapor deposition polymerization. The other side of the membrane is further hydrophilized with PDA *via* mussel-inspired deposition. These two procedures above are aimed at constructing both photothermal function and asymmetric wettability for Janus photothermal membranes. UV-Vis spectroscopy and *in situ* temperature measurement under a simulated solar irradiation are used to investigate photothermal conversion capability of the Janus photothermal membranes. The total efficiency of solar-vapor generation can be assessed by recording the water weight loss in real time.

### 4.2. Reagents and Materials

Commercially available products of PVDF with different molecular weights were obtained from Solvay Solexis (Belgium) (*M*_*n*_ = 110,000 g/mol, Solef 6010) and from Shanghai 3F New Materials Co., Ltd. (China) (*M*_*n*_ = 500,000 g/mol, FR904) and were dried under a vacuum at 60°C for 6 h before use. DMSO2 (99.97%) was purchased from Dakang Chemicals Co., Ltd. (China). Pyrrole (GC grade) was purchased from Aladdin Chemical Co., Ltd. (China). Ammonium persulfate (APS) (AR grade), CuSO_4_·5H_2_O (AR grade), and H_2_O_2_ (30%) were bought from Sinopharm Chemical Reagent Co., Ltd. (China). Dopamine hydrochloride (98%) was purchased from Sigma-Aldrich (China). Sodium fluorescein (AR grade) was purchased from Macklin Biochemical Co., Ltd. (China). Closed-cell polyurethane foam was bought from Hebei Duken Energy Saving Technology Co., Ltd. (China).

### 4.3. Fabrication of VOPM

The fabrication of VOPM is based on our previously reported method [29]. DMSO2 was used as a crystallizable solvent to prepare a homogenous solution of PVDF. Commercially available PVDF powders of Solef 6010 and FR904 with different molecular weights were mixed at a ratio of 7/3 (*w*/*w*) and dissolved in DMSO2 at 180°C with a polymer concentration of 22.5 wt%. After degassing to remove air bubbles, the solution was then sealed in a preheated (160°C) home-made mold consisting of a stainless-steel plate (thickness = 1 mm) and a glass plate (thickness = 3 mm). A Teflon film with a square opening (10 × 10 cm) and a thickness of 200 *μ*m was inserted between two plates to reserve the polymer solution. The plates were then clamped together by clips tightly. Subsequently, the mold was put in an oven at 160°C for several minutes to maintain a constant temperature. After being taken out from the oven, the mold was vertically placed in a reservoir with the addition of water (30°C) at a rate of 1.7 mm/s. After the full concretion, the newborn membrane was taken out from the mold and immersed into deionized water to extract DMSO2. The membrane was then washed with ethanol and hexane in sequence and dried under a vacuum at 60°C for 24 h.

### 4.4. Preparation of PPy Layer

The as-prepared hydrophobic VOPM was cut into circular pieces and immersed successively in ethanol and in 0.5 M of APS solution for 10 min. After removing the surface liquid by filter papers, the wet membrane was placed (L-surface up) in a sealed container (2 L) which was preheated to 50°C. A beaker filled with 20 *μ*L of pyrrole was then located beside in the container to initiate the CVDP reaction. The PPy-deposited membrane was taken out from the container in 30 min, washed with deionized water, and dried under a vacuum at 60°C for 6 h.

### 4.5. Single-Side Deposition with PDA Coating

The single-side decoration of PPy-coated VOPM was conducted by the mussel-inspired deposition accelerated by CuSO_4_/H_2_O_2_ reported in our previous work [31]. Dopamine hydrochloride (2 mg/mL) and CuSO_4_·5H_2_O (1.25 mg/mL) were dissolved in a Tris buffer solution (pH = 8.5, 50 mM). The PPy-coated VOPM was prewetted with ethanol and then floated on the solution surface. An aqueous solution of H_2_O_2_ (30%) was added into the solution to immediately trigger the oxypolymerization of dopamine. The single-side modified membrane was taken out in a few minutes and washed with deionized water overnight. The resulting Janus membrane was then dried under a vacuum for over 6 h. To measure the modification depth, the Janus membranes were prewetted by 80% ethanol solution and then immersed in an aqueous solution of sodium fluorescein (1.0 mg·mL^−1^) to stain the hydrophilic PDA layer. The membranes were taken out in 5 min and washed with deionized water for the LSCM imaging [33].

### 4.6. Characterization

Field Emission Scanning Electron Microscopy (FE-SEM) images were recorded with Hitachi S-4800 (Japan). UV-Vis spectroscopy in a reflection and transmission model was conducted by a UV-2450 spectrometer equipped with an integration sphere (SHIMADZU, Japan). The surface wettability was assessed by the measurement of WCA on a Meter A-200 system (MAIST Vision Inspection & Measurement Co. Ltd., China). The surface temperatures of samples were recorded and analyzed by an iPhone equipped with a thermal infrared camera accessory (FLIR ONE PRO, Flir System. Inc., USA). The concentrations of ions in seawater and condensed water were measured by ICP (Varian 730-ES). The seawater was diluted 5000 times to meet the measuring range of the ICP testing.

### 4.7. Water Evaporation Performance Measurement

The simulated solar irradiation was realized with a Xenon lamp (PL XQ500W, Changzhou Hongming Instrument Technology Co., Ltd., China). A glass baker was filled with water to a height of about 7 cm. A circular sample of membrane with a diameter of 4 cm was then placed on the water surface. The simulated sunlight from the lamp perpendicularly irradiated, and the distance between the membrane and the Xenon lamp was strictly controlled to obtain an intensity of solar irradiation of 1 kW · m^−2^ with the help of a solar power meter. The foam was also cut into a circular sample with a diameter of 4 cm and was wrapped with an absorbent paper. The water content under the composite device was reduced to make sure the intensity of solar irradiation to the membrane surface equaled to 1 kW · m^−2^. The mass change of the system was recorded every 10 min by an electronic balance. All the data were recorded at ambient temperature of 18 ± 2°C and relative humidity of 60 ± 10%. The evaporation rate ($\dot{m}$) was calculated by Equation (3):

(3) $$\begin{array}{c} \dot{m}=\frac{\Delta m}{S\times \Delta t} \end{array}$$

where Δ*m* is the mass change of the system, *S* is the surface area of the membrane, and Δ*t* is the irradiation time. The durability of the membrane was evaluated by 10 cyclic tests of evaporation. Each cycle included one-sun irradiation for 1 h and refreshing with deionized water for 0.5 h.

## Figures and Tables

**Table 1:** Solar vapor generation performances of different membranes under one-sun irradiation (*C*_opt_ = 1) reported in the literatures.

Materials	Morphology	Mode	Light absorbance	Evaporation rate	Solar-vapor conversion efficiency	Ref.
			(%)	(kg·m^−2^·h^−1^)	(%)	
AAO loaded with Al nanoparticles	Vertically aligned pores	Direct contact	96.0	0.93	57.7	[17]
CuS-coated PE membrane	Interconnected macropores	Direct contact	93.0	1.02	63.9	[21]
Hierarchical copper-silicon nanowire porous membrane	Interconnected macropores	Direct contact	93.8	0.81	50.9	[34]
		With thermal insulator	93.8	1.37	86.0	
PPy-coated hydrophilic PVDF membrane	Interconnected macropores	Direct contact	93.0	0.92	54.3	[18]
		Folded into cones	99.2	1.70	93.8	
Electrospinning CB/PMMA-PAN Janus absorber	Interconnected macropores	Direct contact	97.0	0.92	51.0	[15]
		With thermal insulator	97.0	1.30	72.0	
Janus VOPM	Vertically aligned pores	Direct contact	97.0	1.08	62.8	This work
		With thermal insulator	97.0	1.58	90.2	

## Data Availability

All data needed to evaluate the conclusions in the paper are present in the paper and the Supplementary Materials. Additional data related to this paper may be requested from the authors.

## Supplementary Materials

Supplementary Materials Figure S1: (a) ATR-FTIR and (b) XPS spectra of nascent VOPM and Janus VOPM with L- and S-surface for the detection. Figure S2: (a) WCA on the L- and S-surface of nascent VOPM, PPy-coated VOPM, and Janus VOPM (deposition time for PDA coating is 20 min); (b) WCA on the L- and S-surface of Janus VOPM as a function of deposition time. Figure S3: 3D LSCM images of Janus VOPMs constructed with various deposition times of PDA (from 0 min to 60 min). Figure S4: (a) diffuse reflectance spectra and (b) transmission spectra of nascent VOPM and differently treated VOPMs. Figure S5: UV-Vis absorption spectra of PPy-coated VOPM with S- and L-surface towards light, respectively. The inserts are schematic illustrations to the multiscattering effect of cone-like pores. Figure S6: schematic illustration to the setup for the measurement of evaporation rate in a real time under a simulated solar source. Figure S7: water evaporation rates of Janus VOPMs with different deposition time of PDA under one-sun illumination. Figure S8: evaporation rate variations of a Janus VOPM with a thermal insulator under 10 cycles of solar desalination for seawater (the condition of one-sun irradiation for 1 h is used for each cycle). Figure S9: (a) weight loss and (b) evaporation rate of water as a function of time using a Janus VOPM with a thermal insulator for the desalination of seawater and NaCl solution (20 wt%) under one-sun illumination. Figure S10: weight loss and evaporation rate of water as a function of time using a Janus VOPM without a thermal insulator for the desalination of NaCl solution (20 wt%) under one-sun illumination. Table S1: surface element compositions (calculated from XPS results) of nascent VOPM and Janus VOPM with L- and S-surface for the detection.
