# Supplementary material for: Janus Poly(Vinylidene Fluoride) Membranes with Penetrative Pores for Photothermal Desalination
Source: Research (Wash D C). 2020 Mar 3;2020:3241758. doi: 10.34133/2020/3241758 (PMC7072121; doi:10.34133/2020/3241758)
Supplement: Supplementary Materials — Figure S1: (a) ATR-FTIR and (b) XPS spectra of nascent VOPM and Janus VOPM with L- and S-surface for the detection. Figure S2: (a) WCA on the L- and S-surface of nascent VOPM, PPy-coated VOPM, and Janus VOPM (deposition time for PDA coating is 20 min); (b) WCA on the L- and S-surface of Janus VOPM as a function of deposition time. Figure S3: 3D LSCM images of Janus VOPMs constructed with various deposition times of PDA (from 0 min to 60 min). Figure S4: (a) diffuse reflectance spectra and (b) transmission spectra of nascent VOPM and differently treated VOPMs. Figure S5: UV-Vis absorption spectra of PPy-coated VOPM with S- and L-surface towards light, respectively. The inserts are schematic illustrations to the multiscattering effect of cone-like pores. Figure S6: schematic illustration to the setup for the measurement of evaporation rate in a real time under a simulated solar source. Figure S7: water evaporation rates of Janus VOPMs with different deposition time of PDA under one-sun illumination. Figure S8: evaporation rate variations of a Janus VOPM with a thermal insulator under 10 cycles of solar desalination for seawater (the condition of one-sun irradiation for 1 h is used for each cycle). Figure S9: (a) weight loss and (b) evaporation rate of water as a function of time using a Janus VOPM with a thermal insulator for the desalination of seawater and NaCl solution (20 wt%) under one-sun illumination. Figure S10: weight loss and evaporation rate of water as a function of time using a Janus VOPM without a thermal insulator for the desalination of NaCl solution (20 wt%) under one-sun illumination. Table S1: surface element compositions (calculated from XPS results) of nascent VOPM and Janus VOPM with L- and S-surface for the detection. [file 3241758.f1.docx]

Supplementary Materials

Janus Poly(vinylidene fluoride) Membranes with Penetrative Pores for Photothermal Desalination

Hao-Hao Yu^§^, Lin-Jiong Yan^§^, Ye-Cheng Shen, Si-Yu Chen, Hao-Nan Li, Jing Yang* and Zhi-Kang Xu*

MOE Key Laboratory of Macromolecular Synthesis and Functionalization, and Key Laboratory of Adsorption and Separation Materials & Technologies of Zhejiang Province, Department of Polymer Science and Engineering, Zhejiang University, Hangzhou 310027, China

* Correspondence should be addressed to Jing Yang: jing_yang@zju.edu.cn; and Zhi-Kang Xu: [xuzk@zju.edu.cn](mailto:xuzk@zju.edu.cn).

**^§^**These authors contributed equally to this work.


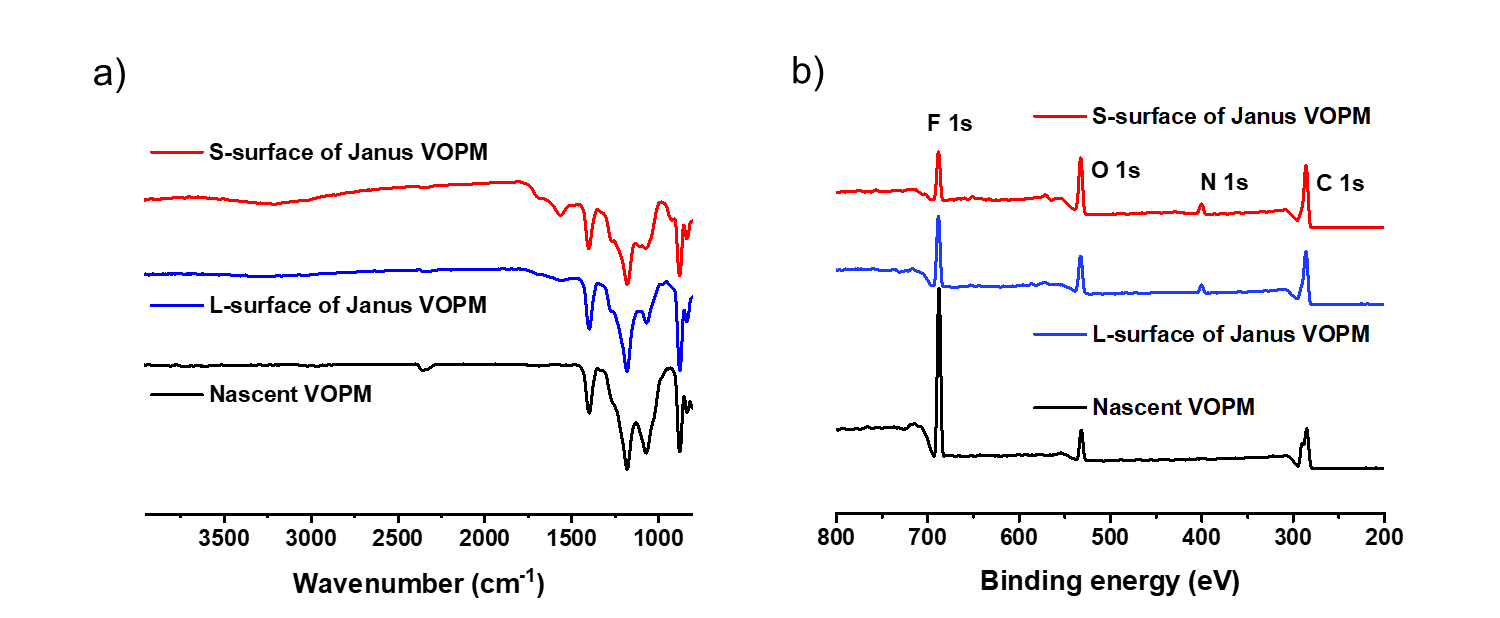


**Figure S1.** (a) ATR-FTIR and (b) XPS spectra of nascent VOPM and Janus VOPM with L- and S-surface for the detection.

**Table S1.** Surface element compositions (calculated from XPS results) of nascent VOPM and Janus VOPM with L- and S-surface for the detection.

|  | C /% | O /% | N /% | F /% |
| --- | --- | --- | --- | --- |
| Nascent VOPM | 49.54 | 10.19 | 1.68 | 38.59 |
| L-surface of Janus VOPM | 60.91 | 14.24 | 4.76 | 20.09 |
| S-surface of Janus VOPM | 60.94 | 18.52 | 6.84 | 13.71 |

For the S-surface coated with PDA, the wide absorption peak centered at 3300 cm^-1^ is assigned to the stretching vibration of O-H and N-H groups of PDA. The other two peaks at 1560 cm^-1^ and 1680 cm^-1^ correspond to the stretching vibration of benzene skeleton and C=N group of PDA, respectively.

For the L-surface coated with PPy, the absorption band of N-H stretching vibration at 3350 cm^-1^ is evident. Another absorption peak at 1560 cm^-1^ is due to the breathing vibration of aromatic skeleton of PPy.

The XPS results show that F element contents of both S- and L-surface of Janus VOPM are significantly lower than those of nascent VOPM. Meanwhile, N element can be detected for Janus VOPM, indicating the formation of PDA or PPy coating.


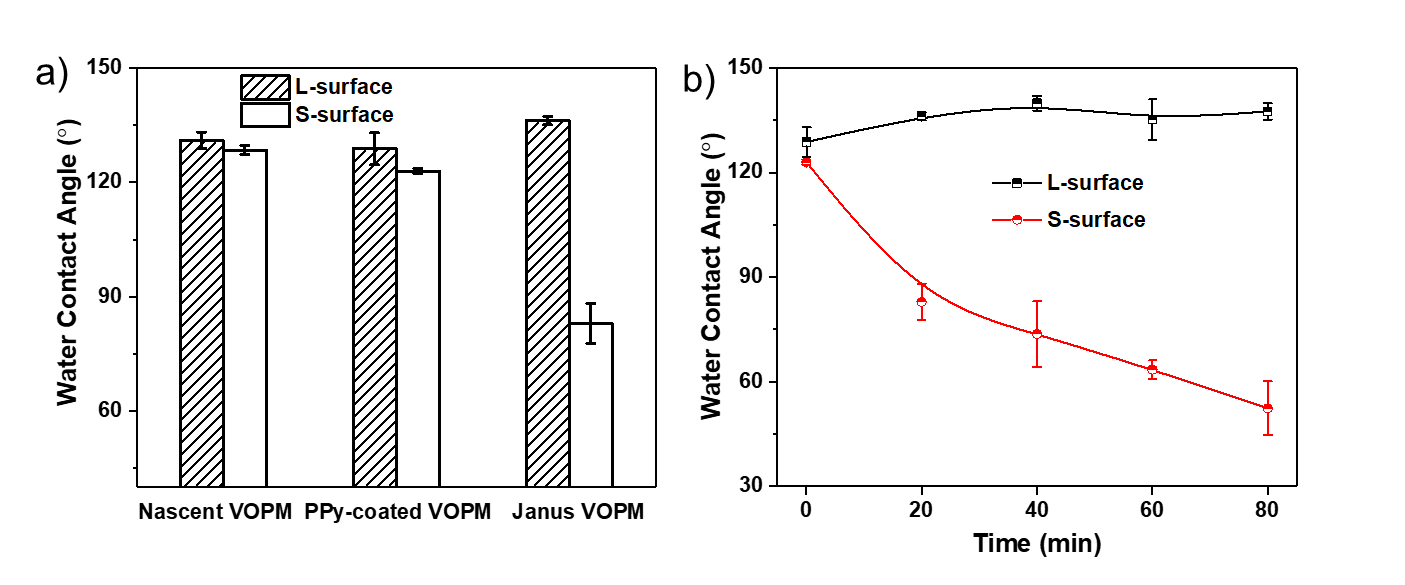


**Figure S2.** (a) WCA on the L- and S-surface of nascent VOPM, PPy-coated VOPM and Janus VOPM (deposition time for PDA coating is 20 min); (b) WCA on the L- and S-surface of Janus VOPM as a function of deposition time.


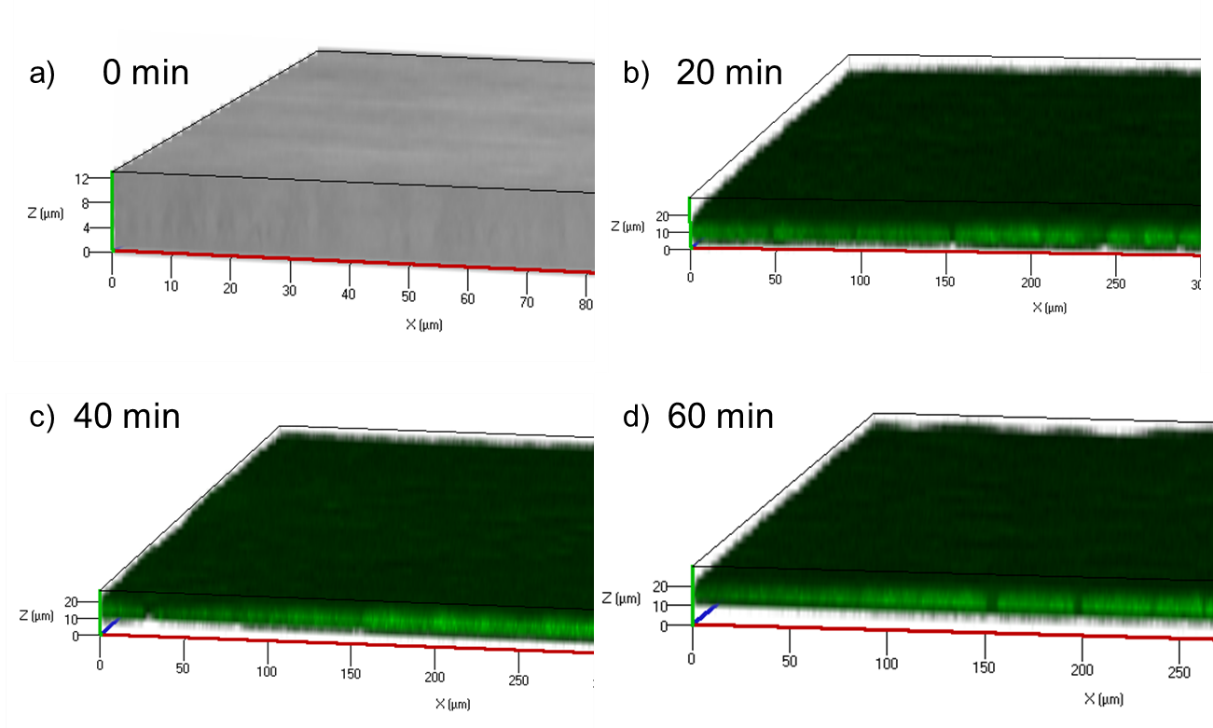


**Figure S3.** 3D LSCM images of Janus VOPMs prepared with various deposition time of PDA (from 0 min to 60 min).


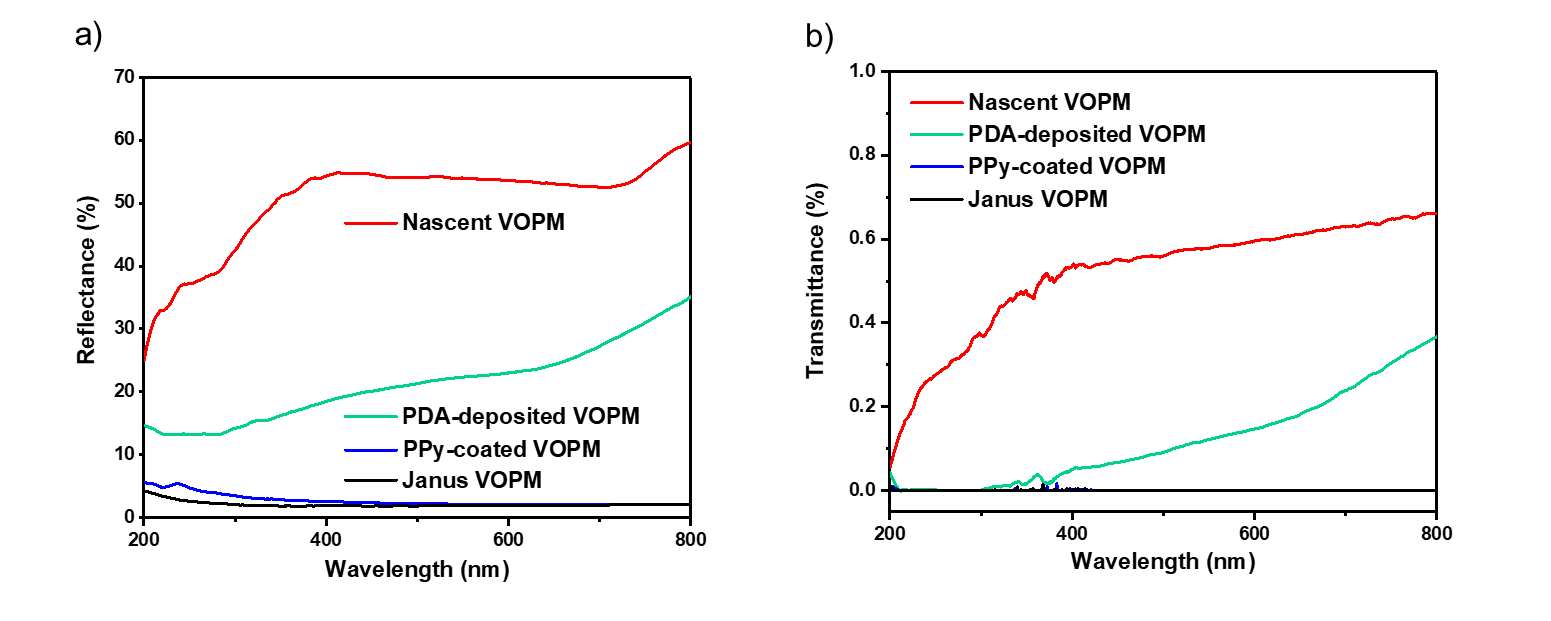


**Figure S4.** (a) Diffuse reflectance spectra and (b) transmission spectra of nascent VOPM and differently treated VOPMs.


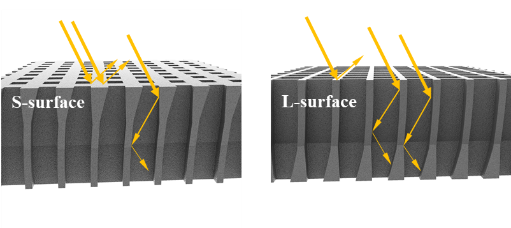


**Figure S5.** UV-Vis absorption spectra of PPy-coated VOPMs with S- and L-surface towards light, respectively. The inserts are schematic illustrations to the multi-scattering effect of cone-like pores.


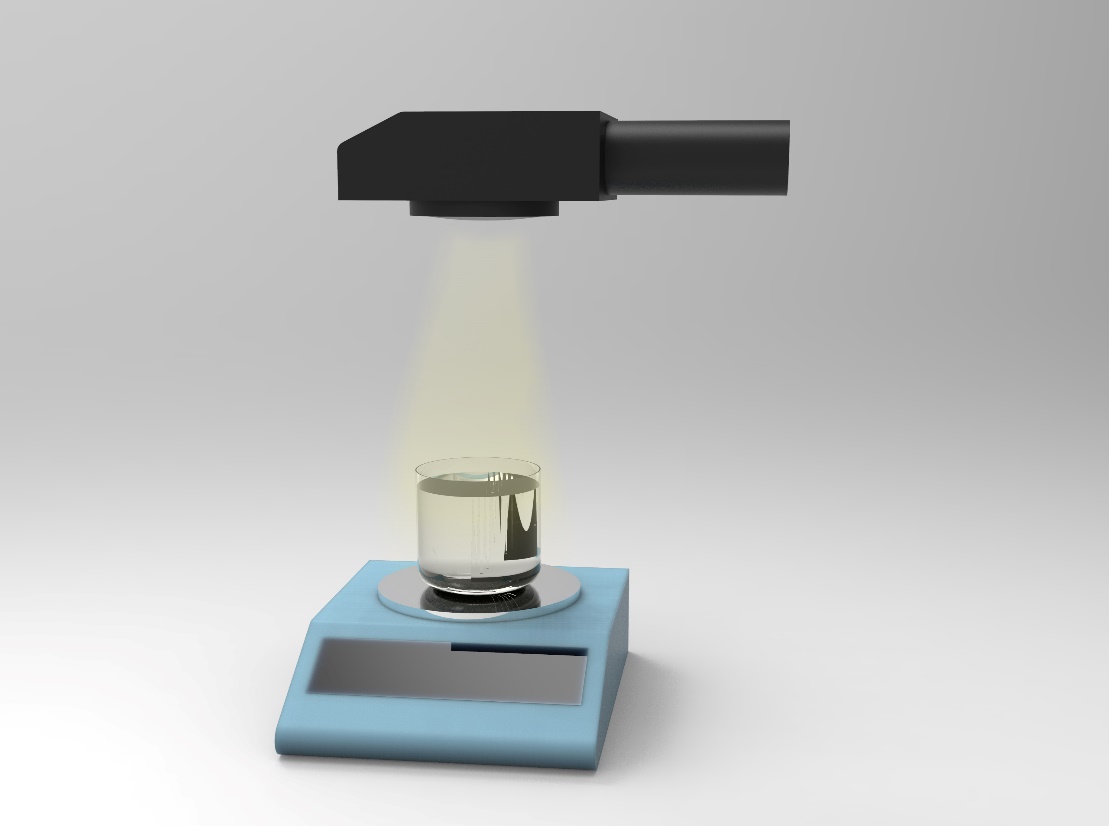


**Figure S6.** Schematic illustration to the setup for the measurement of evaporation rate in a real time under a simulated solar source.

**Figure S7.** Water evaporation rates of Janus VOPMs with different deposition time of PDA under one sun illumination.

**Figure S8.** Evaporation rate variations of a Janus VOPM with a thermal insulator under 10 cycles of solar desalination for seawater (the condition of one-sun irradiation for 1 h is used for each cycle).


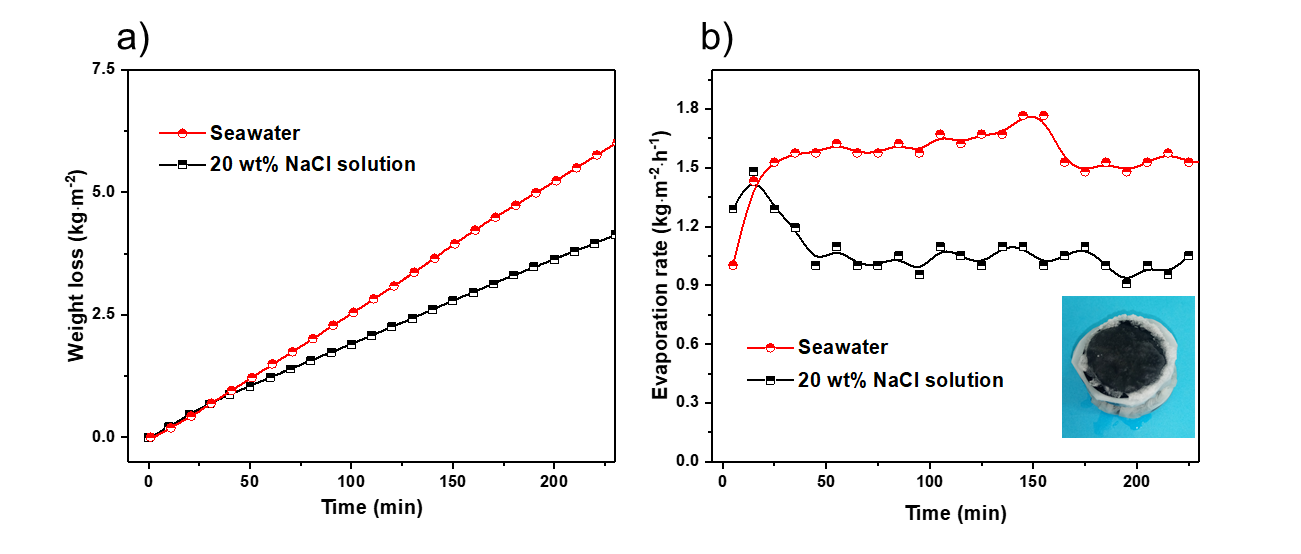


**Figure S9.** (a) Weight loss and (b) evaporation rate of water as a function of time using a Janus VOPM with a thermal insulator for the desalination of seawater and NaCl solution (20 wt%) under one sun illumination.

**Figure S10.** Weight loss and evaporation rate of water as a function of time using a Janus VOPM without a thermal insulator for the desalination of NaCl solution (20 wt%) under one sun illumination.
